# Vitamin D and Chronic Pain in Immigrant and Ethnic Minority Patients—Investigation of the Relationship and Comparison with Native Western Populations

**DOI:** 10.1155/2010/753075

**Published:** 2009-10-19

**Authors:** Sebastian Straube, R. Andrew Moore, Sheena Derry, Ernst Hallier, Henry J. McQuay

**Affiliations:** ^1^Department of Occupational and Social Medicine, University of Göttingen, Waldweg 37 B, 37073 Göttingen, Germany; ^2^Pain Research, Nuffield Department of Anaesthetics, University of Oxford, John Radcliffe Hospital, Level 6 West Wing, Oxford OX3 9DU, UK

## Abstract

Vitamin D deficiency has been implicated in chronic pain. Immigrant and ethnic minority populations have been shown to have lower vitamin D levels than native Western populations and often to be vitamin D deficient. This systematic review investigates the relationship between vitamin D and chronic pain in immigrant and ethnic minority populations. Included were studies reporting on 25-OH vitamin D levels in immigrant/ethnic minority populations affected by chronic pain, and/or reporting on the treatment of chronic pain with vitamin D preparations in such populations. We found that 25-OH vitamin D levels were low and often deficient in immigrant/ethnic minority populations. Vitamin D levels depended on the latitude of the study location and hence sunlight exposure. There was insufficient evidence to reach a verdict on the value of treating chronic pain in immigrant/ethnic minority patients with vitamin D preparations because the studies were few, small, and of low quality.

## 1. Introduction

Chronic pain is among the many conditions that have recently been associated with vitamin D deficiency [1]. A number of studies have suggested a link between low levels of vitamin D and higher incidence of chronic pain [2–4]. Furthermore, indirect evidence that vitamin D levels are important in pain is provided by the association of latitude and season of the year with pain [5–7]. We have recently published a systematic review on the role of vitamin D in chronic pain [8]. It concluded that the evidence did not, on balance, support the hypothesis that 25-OH vitamin D deficiency played a role in chronic painful conditions nor that vitamin D supplementation is a useful treatment for chronic pain.

It is suggested that immigrants and members of ethnic minorities are especially at risk of 25-OH vitamin D deficiency due to darker skin color, low sun exposure, diet, and traditional dress (veiled women, for example). A number of studies show that such populations have lower vitamin D levels and higher prevalence of vitamin D deficiency than native Western populations [9–12]. Other work suggests that there is an excess of widespread pain in immigrant communities along with lower levels of vitamin D [13, 14] and that low levels of vitamin D in ethnic minority populations correlate with musculoskeletal pain [15].

Vitamin D and its roles in health and disease have recently received great interest both in the scientific community [1] and in the popular press [16]. Many tissues express vitamin D receptors and it is not surprising that a physiological role for vitamin D has been proposed in a number of organs and organ systems, not limited to the skeleton. Vitamin D deficiency likewise has been implicated in a number of diseases ranging from autoimmune disease and diabetes to cardiovascular disease, various cancers, and chronic pain [1]. A meta-analysis even suggested reduced all-cause-mortality with vitamin D supplementation [17]. This stands in contrast to the antioxidant vitamins A and E, where widespread supplementation has been questioned after a review suggested a possible increase in mortality rates [18].

Some experts advocate limited and sensible sun exposure and vitamin D supplementation [1] in order to ensure adequate blood levels of 25-OH vitamin D. Excessive dietary supplementation can lead to vitamin D intoxication [19], and excessive sun exposure increases the risk of skin cancers. Skin cancers are a significant health problem and, although cases of vitamin D intoxication have been reported only infrequently, they could become more common with widespread use of vitamin D supplements. Before the use of vitamin D supplements can be advocated, the evidence for health benefits and harm needs to be assessed rigorously in each of the proposed areas of benefit.

If there were a link between 25-OH vitamin D deficiency and chronic pain, one might expect to find an inverse association between pain and 25-OH vitamin D levels and a demonstrable benefit of vitamin D supplementation with regard to pain outcomes in interventional studies. Our previous systematic review [8] did not support either hypothesis for the overall population. As immigrants and members of ethnic minorities are more severely affected by 25-OH vitamin D deficiency, and where any association between vitamin D and chronic pain should be stronger, it is important to re-examine the issue in this particular population subgroup.

## 2. Material and Methods

We searched Medline (PubMed) using various search terms for vitamin D (vitamin D; vitamin D_2_; vitamin D_3_; 1-alpha hydroxyvitamin D_3_; 1-alpha hydroxycalciferol; 1,25-dihydroxyvitamin D_3_; 1,25-dihydroxycholecalciferol; 25 hydroxycholecalciferol; 25-hydroxyvitamin D; alfacalcidol; calcidiol; calcitriol; calcifediol; calciferol; ergocalciferol; cholecalciferol; and spelling variations thereof) and “pain*.” The last search was conducted on February 9, 2009. The limits in PubMed were set to “humans.” Because electronic searching commonly does not reveal all relevant studies (particularly observational studies), we also searched through the references of retrieved articles and relevant review articles for additional publications. We sought reports of any kind of clinical study published in any language and included all studies that reported on ethnic minority or immigrant populations with chronic pain conditions.

For inclusion in this review studies needed to be conducted in ethnic minority or immigrant subjects with chronic pain, to give mean values for 25-OH vitamin D levels in these populations, and/or to investigate pain outcomes after vitamin D supplementation. We accepted any dose and regimen of vitamin D supplementation. We included only full publications (no abstracts). We did not include individual case reports. Vitamin D requirements differ in children and adults so that a comparison of vitamin D levels between populations of children and adults would not be instructive. We, therefore, restricted the scope of this review to studies in adult populations. For comparison, with the general Western populations, we took data from our recent systematic review [8].

We deliberately defined chronic pain in the broadest possible sense and accepted any kind of vitamin D therapy in order to be inclusive and uncover any association. Osteomalacia can result from vitamin D deficiency in adults, and manifest as musculoskeletal pain. The link between vitamin D deficiency and pain in osteomalacia is well established, and in frank osteomalacia, the case may be obvious. There is the possibility that “subclinical” osteomalacia might manifest as musculoskeletal pain. In fact, there might well be a spectrum, from hypovitaminosis D, through to frank osteomalacia, with subclinical osteomalacia manifesting as musculoskeletal pain in some individuals. On the other hand, vitamin D deficiency might potentially cause chronic pain by some other mechanism and changes in markers of bone metabolism might be coincidental. We did not exclude studies of patients with osteomalacia; but in order to be included in this review, all subjects in the study population needed to have chronic pain.

We extracted information about study design, study location, condition and population, patient characteristics, mean 25-OH vitamin D levels, types and doses of vitamin D preparations used, study duration, pain outcomes, and adverse events. We extracted information for patients in “pain” and healthy “control” groups when their 25-OH vitamin D levels were reported, or when they were treated with vitamin D preparations. Where possible, we also did this for nonimmigrant populations in the same study, for the sake of comparison with immigrant/ethnic minority patients. Guidelines for quality of reporting of meta-analyses were followed where appropriate [20].

## 3. Results

We identified seven relevant studies; details are in Table 1. There were no randomised controlled trials. Only two small studies investigated treatment with vitamin D [21, 22]; both were case series. Five studies were purely observational with no intervention [3, 23–26]. The total number of immigrant or ethnic minority patients in “pain” and “control” groups was 431. Six of the seven studies were of musculoskeletal pain; another one [26] included subjects with any kind of chronic persistent pain.

The two case series investigating treatment with vitamin D [21, 22] together had 17 patients. De Torrenté de la Jara et al. [22] stated that for most of their patients treatment consisted of intramuscular injections of 300 000 IU of cholecalciferol at monthly intervals and an ongoing course of oral calcium (1000 mg) and cholecalciferol (20 *μ*g). Nellen et al. [21] did not specify treatment details other than that it consisted of vitamin D and calcium. All 17 patients were reported to improve with vitamin D treatment. According to De Torrenté de la Jara et al. [22] symptoms (musculoskeletal pain) disappeared within three months in 10/11 patients and within seven months in all. Nellen et al. [21] found that all of their patients became symptom free within three months. Neither study used validated scales for the assessment of pain or commented on adverse events.

All but one noninterventional study [26] reported mean 25-OH vitamin D levels in subjects with chronic pain in the deficiency range below 20 ng/mL [1]. One study [3] found lower levels of 25-OH vitamin D in eight Australian Aborigines affected by chronic pain than in eight Aboriginal controls. Another in the UK [25] found similar 25-OH vitamin D levels in 127 South Asians affected by chronic widespread pain as in South Asians diagnosed with specific rheumatic diseases. A comparison of 83 immigrants with musculoskeletal pain in Minnesota with 67 nonimmigrants with musculoskeletal pain found slightly higher levels of 25-OH vitamin D in the immigrants [24], though both “immigrant” and “nonimmigrant” populations were ethnically diverse.

**Figure 1:**
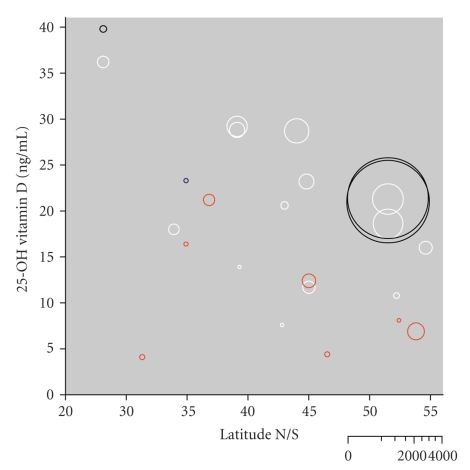
25-OH vitamin D levels in pain subjects and nonpain controls according to latitude (northern or southern hemisphere). White circles indicate pain subjects from the general Western population; black circles indicate nonpain (control) subjects from the general population. Red circles show data for pain subjects from immigrant/ethnic minority populations and the blue circle is from an ethnic minority nonpain control population. The size of the symbol is proportional to the size of the study populations (inset logarithmic scale).

Figure 1 compares 25-OH vitamin D levels in immigrants and ethnic minority populations from this review with data from our recent review on 25-OH vitamin D levels in the general population [8]. Overall, we found the expected dependence of 25-OH vitamin D level on latitude of study location (sunlight) and lower levels of 25-OH vitamin D in immigrant/ethnic minority populations with chronic pain compared with patients from the general population with chronic pain.

## 4. Discussion

On the one hand, there is a considerable evidence that 25-OH vitamin D levels are lower in immigrant and ethnic minority than in native Western populations [9–12], and circumstantial evidence that low levels of 25-OH vitamin D are implicated in chronic pain [1–8, 27, 28]. On the other hand, this systematic review uncovered very little evidence comparing average levels of 25-OH vitamin D between “pain” and “control” patients from immigrant/ethnic minority populations or investigating treatment with vitamin D for chronic pain in such populations. Despite using a broad inclusive search strategy, there was a dearth of evidence.

What little evidence we found was of low quality. There were no randomised controlled trials. Treatment studies were too small and reported insufficient detail on the vitamin D preparations used and on outcome measures to allow meaningful conclusions to be drawn.

All but one study found average levels 25-OH vitamin D to be deficient. In isolation, this is hard to interpret. The only study [24] comparing immigrants with nonimmigrants did so in a way that both “immigrant” and “nonimmigrant” groups were ethnically diverse. South Asians in the UK with chronic widespread pain had similar vitamin D levels to South Asians with specific rheumatic diseases [25]. Subjects with specific rheumatic diseases, however, are an inappropriate control for the question of this review because some of them may well have had chronic pain (which is why data from this group are not included as “controls” in Figure 1). The only study comparing 25-OH vitamin D levels in an ethnic minority group with chronic pain with controls from the same ethnic minority [3] did find lower levels of 25-OH vitamin D in the subjects with pain. However, this study was too small, with 16 subjects, to allow any meaningful interpretation.

Comparing immigrant/ethnic minority populations and general Western populations with chronic pain, we found lower levels of 25-OH vitamin D in immigrant/ethnic minority populations, sometimes very low (Figure 1). This is, however, similar to what is found in populations without chronic pain. Immigrants tend to have lower 25-OH vitamin D levels than nonimmigrants, regardless of whether they suffer from chronic painful conditions. This might indicate independence of 25-OH vitamin D levels from chronic pain; but this is only speculation because the amount of information is so small, and studies were inconsistent in what they measured and reported.

## 5. Conclusions

There is insufficient evidence of high quality to confirm a link between 25-OH vitamin D levels and chronic pain in immigrant and ethnic minority populations. Absence of evidence is not an evidence of absence, and we cannot conclude that vitamin D is not associated with chronic pain, though we can begin to question the strength of any association. The subject is important because vitamin D deficiency is common, and commonly severe in immigrants, and treatment—if indicated—would be easy and cheap. Therefore, the question addressed in this review demands further studies, better studies, and better reporting of study results. We are not alone in thinking that more and better studies on the effect of vitamin D in chronic pain are needed *[*28*]*. What we need are large double-blind randomized controlled trials, conducted in different painful conditions, stratified by baseline 25-OH vitamin D level, with defined interventions and ideally with outcome stratified by post-treatment 25-OH vitamin D level. Given the limited level of knowledge, a placebo group would be necessary to demonstrate causation and effect; 25-OH vitamin D levels ought to be monitored. These randomized controlled trials would need standardized, validated pain outcomes (such as the number of patients achieving at least 50% pain relief), and for an assessment of benefit versus harm would also need to report on adverse events.

## Figures and Tables

**Table 1:** Characteristics of included studies.

Reference	Study type	Location	Condition	Study population	Numbers of immigrant/ethnic minority patients with painful conditions and data on vitamin D levels	Mean 25-OH vitamin D (ng/mL) in immigrant/ethnic minority patients	Outcome of vitamin D treatment
Lowenthal and Shany 1994 [23]	Case series	Beer Sheva, Israel	Osteomalacia (with bone pain)	Bedouin Arab women (an ethnic minority in Israel)	12	4.1	
Nellen et al.1996 [21]	Case series	Amsterdam, Netherlands	Hypovitaminosis D osteopathy (with musculoskeletal pain)	Immigrant women	6	8.1	Symptoms resolved in all patients
Plotnikoff and Quigley 2003 [24]	Cross-sectional study	Minneapolis, Minnesota, USA	Musculoskeletal pain	Immigrant and nonimmigrant patients	83	12.4	
De Torrenté de la Jara et al. 2004 [22]	Case series	Lausanne, Switzerland	Hypovitaminosis D (with musculoskeletal pain)	Female asylum seekers	11	4.4	Pain disappeared in 10/11 within three months and in all patients within seven months
Benson et al. 2006 [3]	Case-control study	Adelaide, Australia	Muscle pain	Australian Aborigines	8	16.4	
Helliwell et al. 2006 [25]	Case-control study	Leeds, UK	Musculoskeletal pain	Patients of South Asian ethnicity	127	6.9	
Bartley 2008 [26]	Cross-sectional study	Auckland, New Zealand	Chronic pain	Ethnic minority and European ethnicity patients	58	21.2	
